# Supplementary material for: Self-assembled organic nanomedicine enables ultrastable photo-to-heat converting theranostics in the second near-infrared biowindow
Source: Nat Commun. 2021 Jan 11;12:218. doi: 10.1038/s41467-020-20566-6 (PMC7801739; doi:10.1038/s41467-020-20566-6)
Supplement: Supplementary file 1 — Supplementary Information [file 41467_2020_20566_MOESM1_ESM.pdf]

Supplementary Information

Self-Assembled Organic Nanomedicine Enables Ultrastable Photo-to-Heat Converting Theranostics in the Second Near Infrared Biowindow

Xiang et al.

Section S1: Materials and Methods

Materials

Boron trifluoride diethyl etherate (BF_3•OEt_2), cyanoacetic acid, N,N-dimethyl-*p*-phenylenediamine, sodium nitrite ($NaNO_2$), and triethylamine (NEt_3) were purchased from Sigma-Aldrich. Annexin V-fluoresceine isothiocyanate/propidium iodide (Annexin V-FITC/PI) apoptosis and necrosis detection kit, calcein acetoxymethyl ester (Calcein-AM)/PI double stain kit, 4',6-diamidino-2-phenylindole (DAPI), and Phalloidin-Alexa Fluor 488 stain kit were acquired from Thermal Fisher Scientific. All chemicals were used without further purifications. Deionized water used in all experiments was prepared by Milli-Q system (Millipore, USA).

Characterizations

NMR spectra were recorded on a Bruker AV-400 spectrometer, and chemical shifts (δ, ppm) were determined by using internal reference tetramethyl silane. High-resolution mass spectrometry (HRMS) was performed on a waters Q-tof Premier MS spectrometer. Transmission electron microscopy (TEM) images were recorded on a JEM-1400 (JEOL) microscope at an acceleration voltage of 100 kV. UV-vis-NIR absorption spectra were recorded on a UV-3600 Shimadzu UV-vis-NIR spectrometer. The hydrodynamic diameter was determined by a Malvern Zetasizer Nano ZS90 dynamic light scattering (DLS) system. The quantitative analysis of elements was conducted by an Agilent 725 inductively coupled plasma-optical emission spectrometer (ICP-OES). The temperature and thermal images were recorded on an infrared thermal imaging instrument (FLIR A325SC camera). 808 nm and 1064 nm lasers (Shanghai Connect Fiber Optics Co.) were used as the irradiation source for photothermal conversion. Confocal laser scanning microscope (CLSM) images were acquired using a FV1000 Olympus confocal laser scanning microscope. Cell apoptosis was assessed by BD LSRFortessa flow cytometry. Photoacoustic images were recorded by a Vevo LAZR photoacoustic imaging system.

Synthesis of BF_2-formazanate (2)

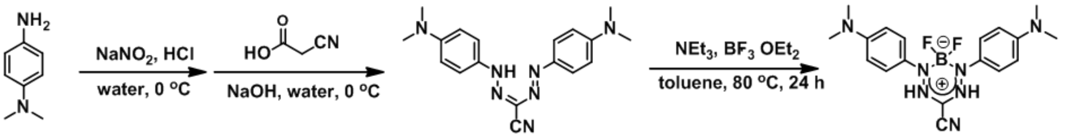

formazan (1) BF₂-formazan (2)

Formazan (1)

Cyanoacetic acid (2.0 g, 24 mmol) and NaOH (11.0 g, 280 mmol) were dissolved in deionized water (500 mL), and the mixture was cooled down to 0 °C and stirred for 30 min. Afterwards, concentrated HCl (12 M, 15 mL) was slowly added to a mixture of N,N-dimethyl-*p*-phenylenediamine (6.5 g, 48 mmol) and deionized water (100 mL), and the solution was stirred at 0 °C for 15 min. Subsequently, $NaNO_2$ aqueous solution (2.4 M, 20 mL) cooled to 0 °C was added dropwise to above N,N-dimethyl-*p*-phenylenediamine solution over 15 min. The obtained red/brown solution was stirred for 10 min, which was added dropwise to the prepared alkaline cyanoacetic acid solution for 15 min. The mixture was stirred for 3 h and neutralized with HCl (1 M). The organic layer was extracted using CH_2Cl_2 and washed with deionized water, and the dark-purple fraction was collected and concentrated to afford dark-purple solid. The crude product was recrystallized from a saturated MeOH solution to afford formazan **1** as a dark-purple microcrystalline solid (1.285 g, 16% yield). ^1H NMR (400 MHz, DMSO-d_6): δ 12.04 (s, 1H), 7.64 (d, J = 9 Hz, 4H), 6.81 (d, J = 9 Hz, 4H), 3.01 (s, 12H). ^{13}C NMR (100 MHz, DMSO-d_6): δ 150.2, 138.0, 125.4, 121.4, 114.1, 112.4, 40.1. HRMS (CI): calculated for $[C_{18}H_{21}N_7]^+$: 335.1858; found: 335.1865.

BF_2-formazanate (2)

Under N_2, formazan **1** (1.03 g, 3 mmol) and NEt_3 (1.5 mL, 10.5 mmol) were added to toluene (180 mL) and the mixture was stirred for 10 min. BF_3•OEt_2 (2.0 mL, 16.8 mmol) was slowly added to the above solution, and the mixture was heated at 80 °C for 24 h. Subsequently, the mixture was cooled down to room temperature, and deionized water was added to the solution. Then, the organic layer was rinsed with deionized water for 3 times, dried over $MgSO_4$ and concentrated to obtain crude product. The crude product was purified using column

chromatography ($CH_3COOC_2H_5/CH_2Cl_2$ = 1:1 (v/v)) to afford a dark-purple solid (0.56 g, 45% yield). ^{1}H NMR (400 MHz, $CDCl_3$): δ 7.82 (d, J = 9.2 Hz, 4H), 6.68 (d, J = 9.4 Hz, 4H), 3.09 (s, 12H). ^{13}C NMR (100 MHz, $CDCl_3$): δ 151.5, 133.7, 124.6, 115.8, 112.4, 112.1, 40.4. ^{19}F NMR (376 MHz, $CDCl_3$): δ -137.4 (q, J = 31.8 Hz). HRMS (CI): calculated for $[C_{18}H_{20}N_7BF_2]^+$: 383.1841; found: 383.1793.

Cell culture

Murine breast cancer 4T1 cells and human breast adenocarcinoma MCF-7 cells were cultured at 37 °C in a humidity atmosphere containing 5% CO_2 in DMEM supplemented with 10% fetal bovine serum (FBS) and 1% penicillin-streptomycin (PS).

Calculation of photothermal conversion efficiency

The photothermal conversion efficiency of Nano-BFF was calculated by monitoring the temperature change of Nano-BFF in aqueous dispersion as a function of time under continuous laser irradiation. When the temperature reached to a plateau, the laser was turned off and the temperature was recorded during the cooling stage until the temperature decreased to the room temperature.

Details are listed as follows:

Based on the total energy balance for this system:

$$\sum_i m_i C_{p,i} \frac{dT}{dt} = Q_s - Q_{loss}$$

where m_i is the mass and $C_{p,i}$ is the heat capacity of system components, respectively. Q_s is the photothermal heat energy input by irradiating Nano-BFF solution with NIR laser, and Q_{loss} is thermal energy lost to the surroundings. When the temperature is the maximum, the system is in balance.

$$Q_s = Q_{loss} = hS\Delta T_{max}$$

where h is heat transfer coefficient, S is the surface area of the container, and ΔT_{max} is the maximum temperature change.

The photothermal conversion efficiency is calculated from the following equation:

$$\eta = \frac{hS\Delta T_{max}}{I(1-10^{-A_{\lambda}})}$$

where I is the laser power and λ is the absorbance of Nano-BFF solution at the wavelength of 1064 nm or 808 nm.

In order to get the hS, a dimensionless driving force temperature, θ is introduced as follows:

$$\theta = \frac{T-T_{surr}}{T_{max}-T_{surr}}$$

where T is the temperature of Nano-BFF solution, T_{max} is the maximum system temperature, and T_{surr} is the initial temperature. A sample system time constant τ_s,

$$\tau_s = \frac{\sum_i m_i C_{p,i}}{hS}$$

Thus,

$$\frac{d\theta}{dt} = \frac{1}{\tau_s}\frac{Q_s}{hS\Delta T_{max}} - \frac{\theta}{\tau_s}$$

When the laser is off, $Q_s = 0$, therefore $\frac{d\theta}{dt} = -\frac{\theta}{\tau_s}$, $t = -\tau_s \ln\theta$. So, hS could be calculated from the slope of cooling time *vs* lnθ. The time constant (τ_s) of heat transfer from Nano-BFF was determined to be 220.42 s and 185.01 s for 808 nm and 1064 nm, respectively (Fig. 2c,g). The ΔT_{max} of Nano-BFF was 25.6 °C and 24.1 °C for 808 nm and 1064 nm laser irradiation, respectively. Therefore, the photothermal conversion efficiency (η) of Nano-BFF was calculated to be 28.6% and 34.3% at 808 nm and 1064 nm, respectively.

Section S2. Additional Supplementary Data and Figures

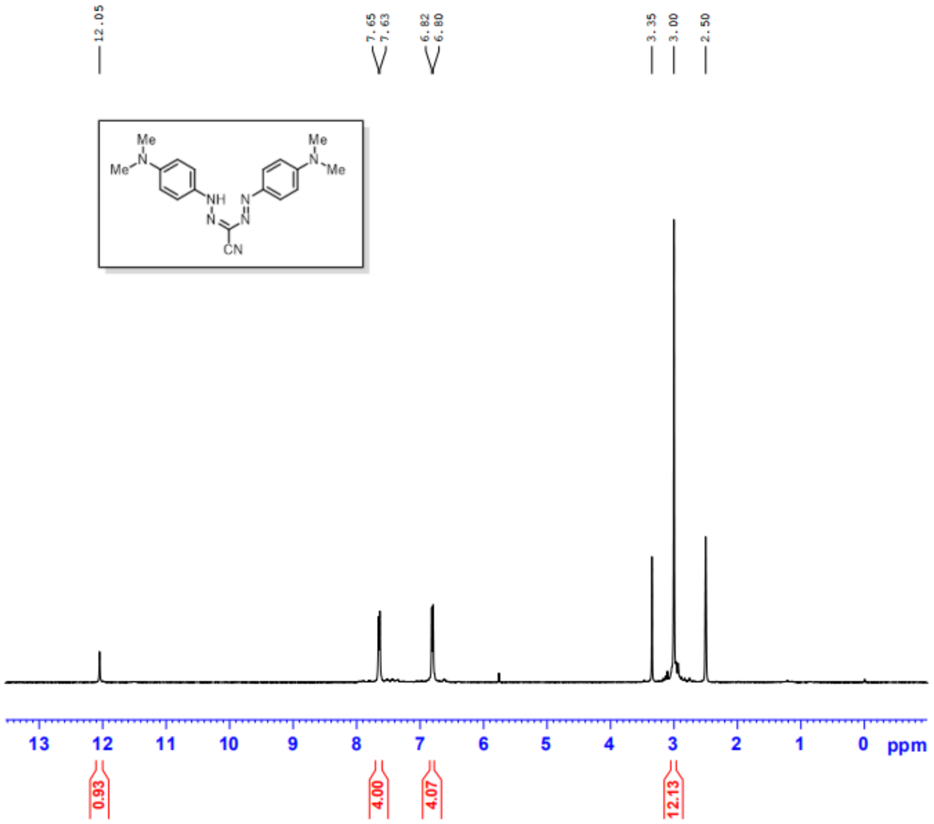

Supplementary Figure 1. ^{1}H NMR spectrum of formazan **(1)**.

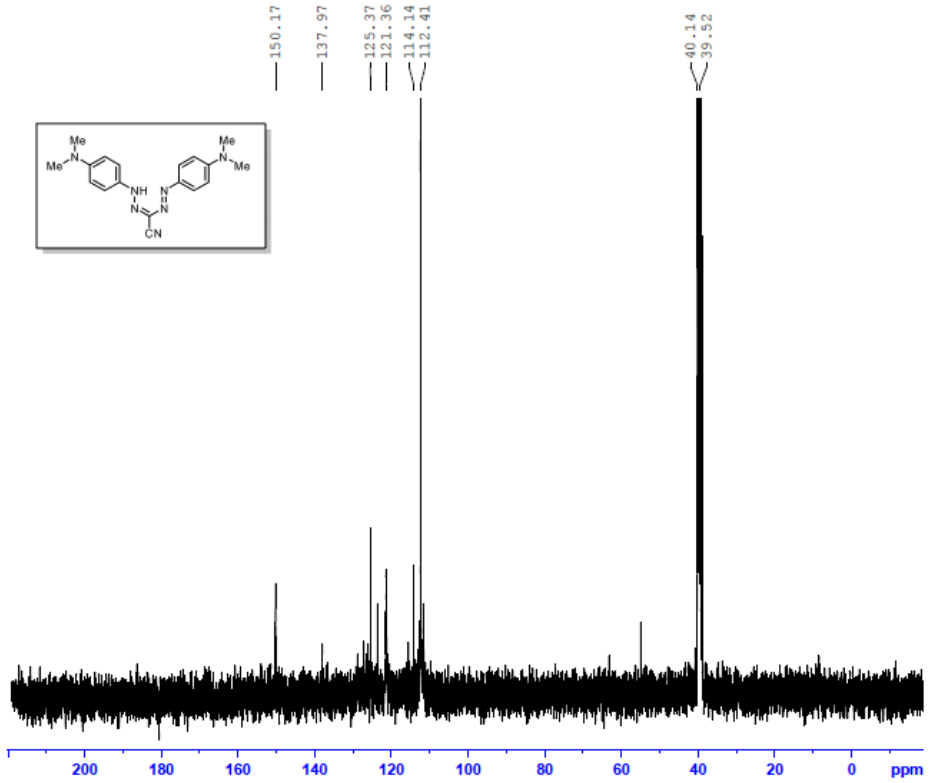

Supplementary Figure 2. ^{13}C NMR spectrum of formazan **(1)**.

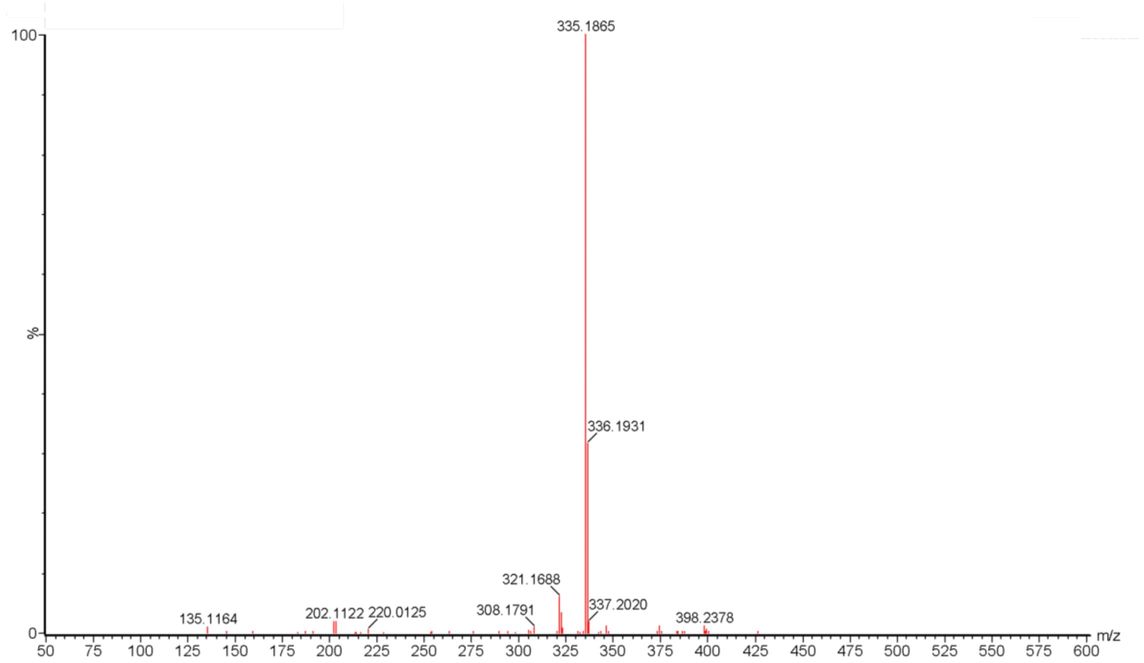

Supplementary Figure 3. HRMS spectrum of formazan **(1)**.

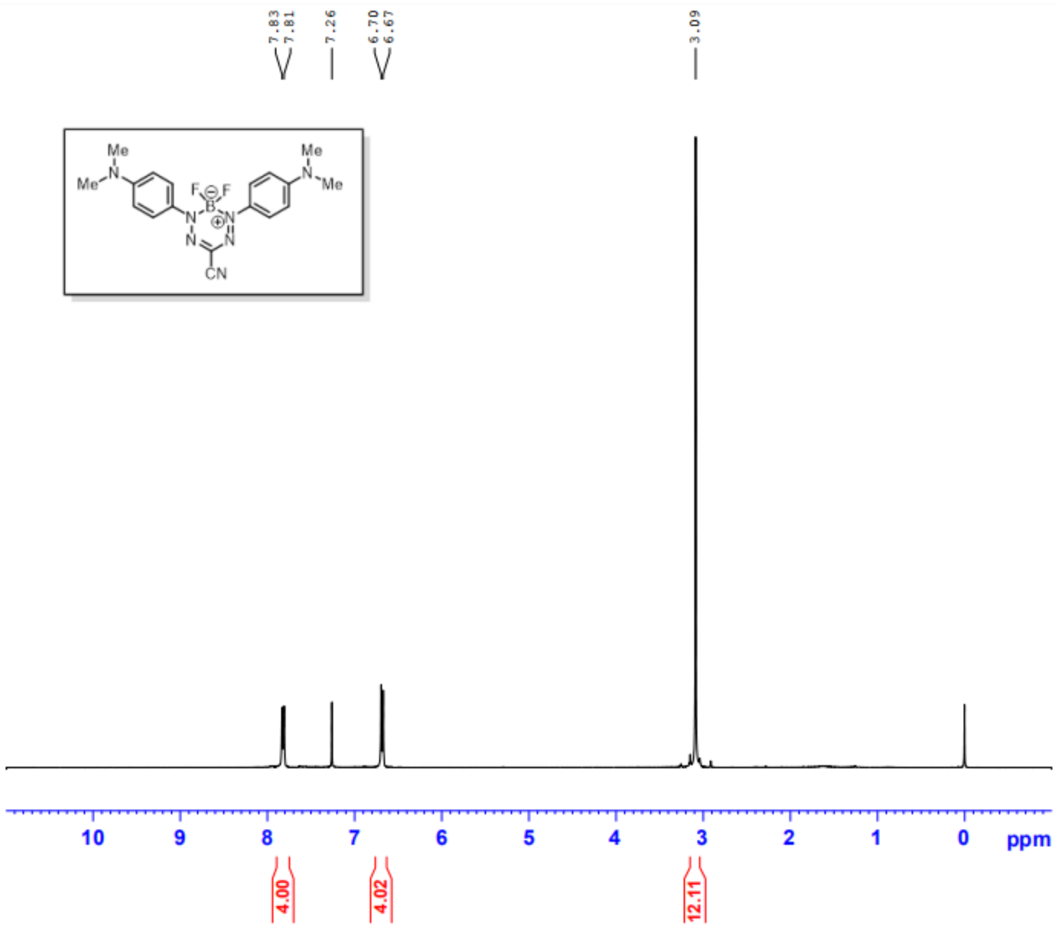

Supplementary Figure 4. ^{1}H NMR spectrum of BF_2-formazanate **(2)**.

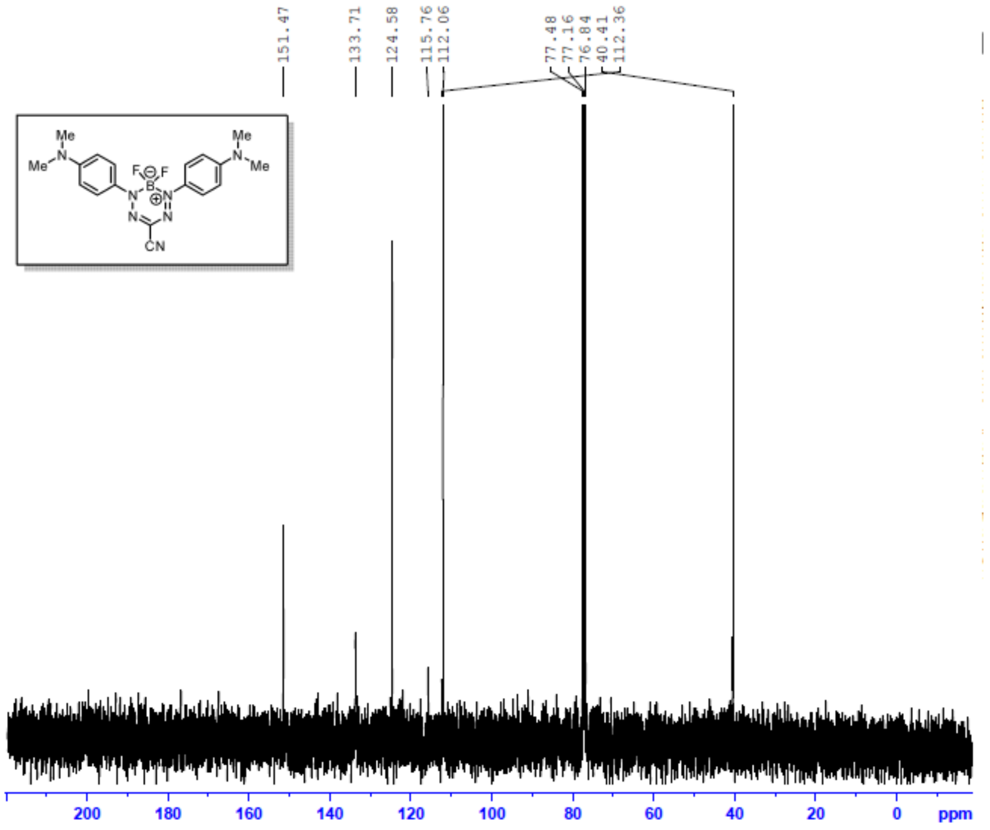

Supplementary Figure 5. ^{13}C NMR spectrum of BF_2-formazanate **(2)**.

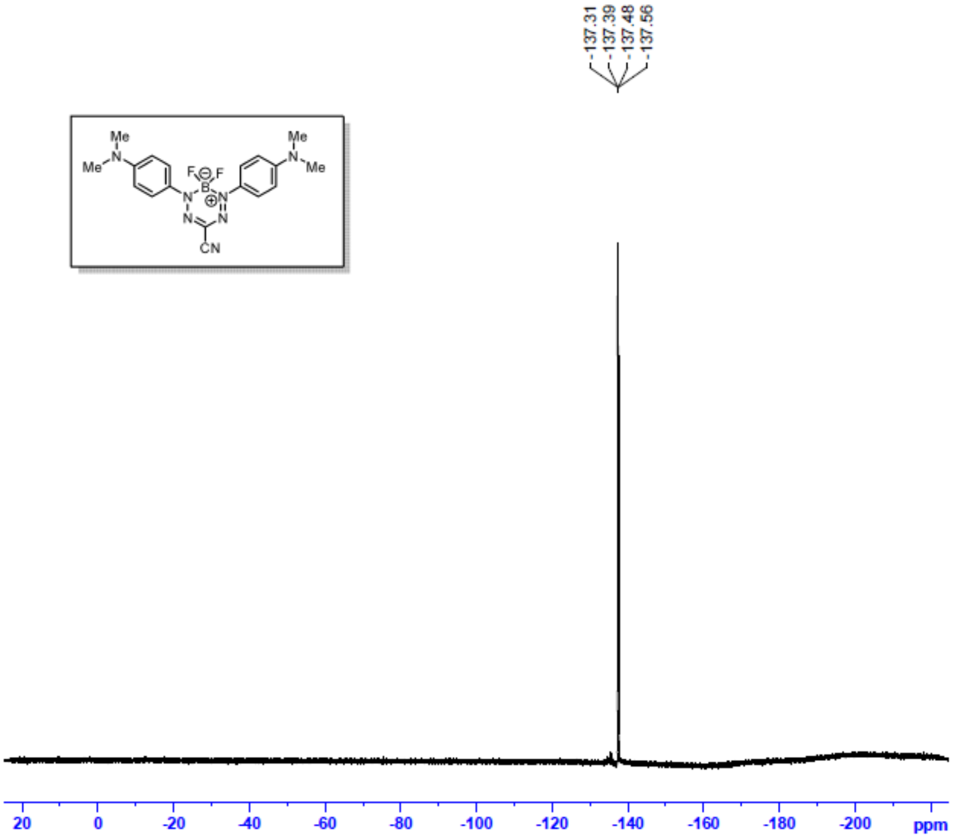

Supplementary Figure 6. ^{19}F NMR spectrum of BF_2-formazanate **(2)**.

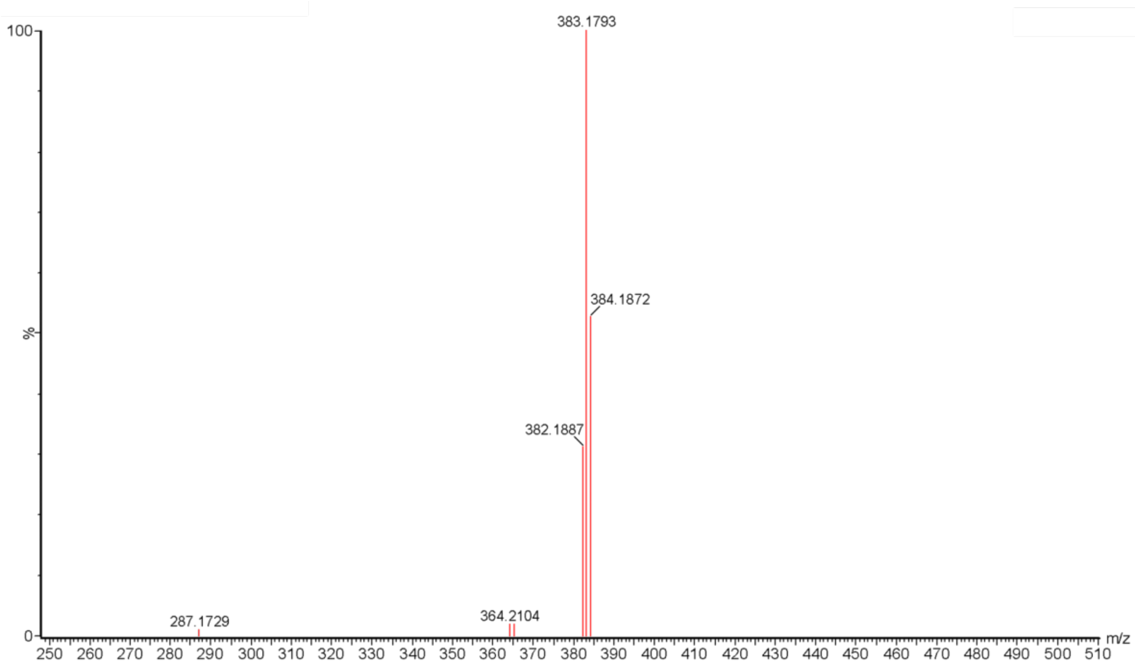

Supplementary Figure 7. HRMS spectrum of BF_2-formazanate **(2)**.

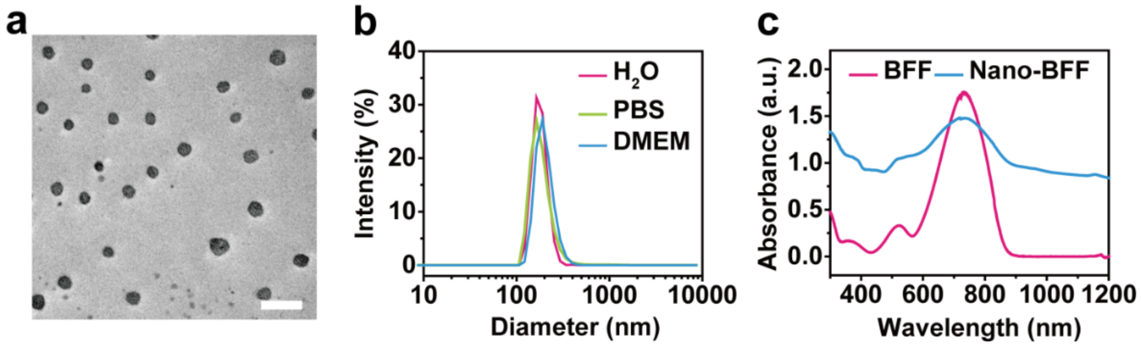

Supplementary Figure 8. Characterization of Nano-BFF. a TEM image (scale bar: 0.5 μm), and **b** hydrodynamic diameters of Nano-BFF. **c** UV-vis-NIR absorption spectra of BFF dissolved in tetrahydrofuran and Nano-BFF dispersed in water, respectively. A representative image of three biological replicates from each group is shown in **a**.

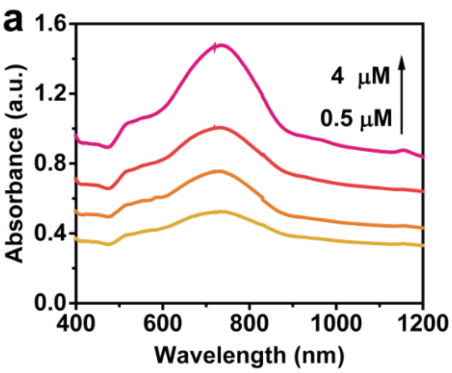

Supplementary Figure 9. Absorption spectra and molar extinction coefficient. a UV-vis-NIR absorption spectra of various concentrations of Nano-BFF. **b** Molar extinction coefficient of Nano-BFF at 808 nm and 1064 nm.

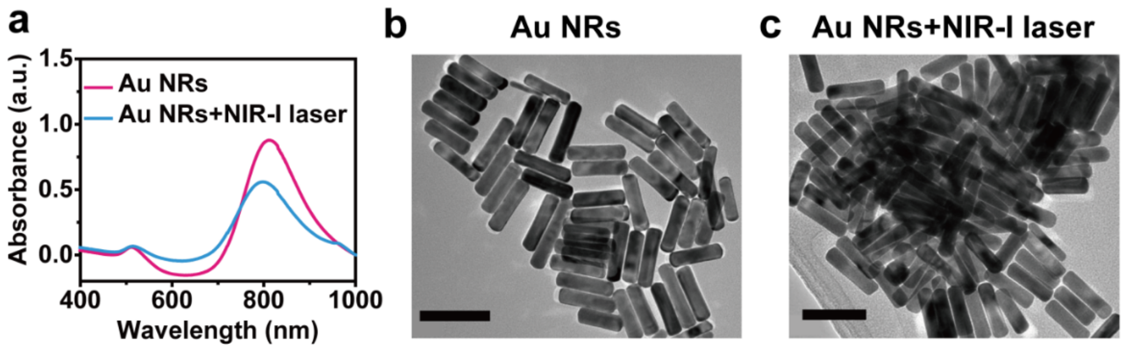

Supplementary Figure 10. Au nanorods (NRs) under continuous NIR-I laser irradiation. a UV-vis-NIR absorption spectra of Au NRs before and after continuous NIR-I laser irradiation for 7 min. **b**,**c** TEM images of **b** Au NRs, and **c** Au NRs after continuous NIR-I laser irradiation for 7 min. Scale bars: 100 μm. A representative image of three biological replicates from each group is shown.

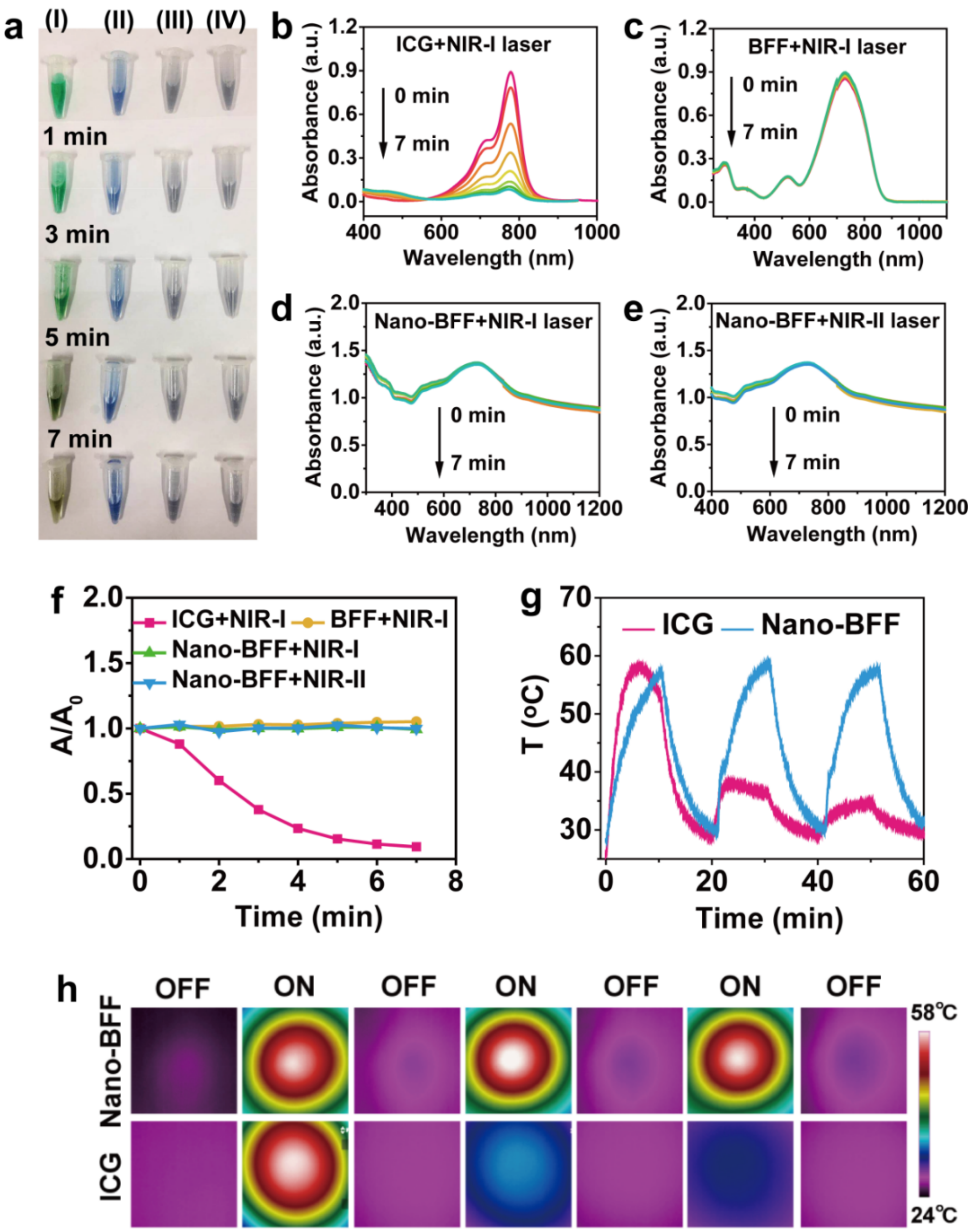

Supplementary Figure 11. Photostability of Nano-BFF. a Photographs of ICG, BFF, and Nano-BFF after repeated laser irradiation for 7 min, respectively. I: ICG + NIR-I laser; II: BFF + NIR-I laser; III: Nano-BFF + NIR-I laser; IV: Nano-BFF + NIR-II laser. **b-d** UV-vis-NIR absorption spectra of **b** ICG, **c** BFF, and **d** Nano-BFF after repeated NIR-I laser irradiation for 7 min. **e** UV-vis-NIR absorption spectra of Nano-BFF under repeated NIR-II laser illumination for 7 min. **f** Plots of relative absorbance intensity of ICG, BFF and Nano-BFF versus irradiation time (n = 3 biologically independent samples). **g** Thermal curves of Nano-BFF and ICG after repeated laser exposure. **h** Thermal images of Nano-BFF and ICG after repeated 808 nm laser exposure (power density: 1 W cm^{-2}).

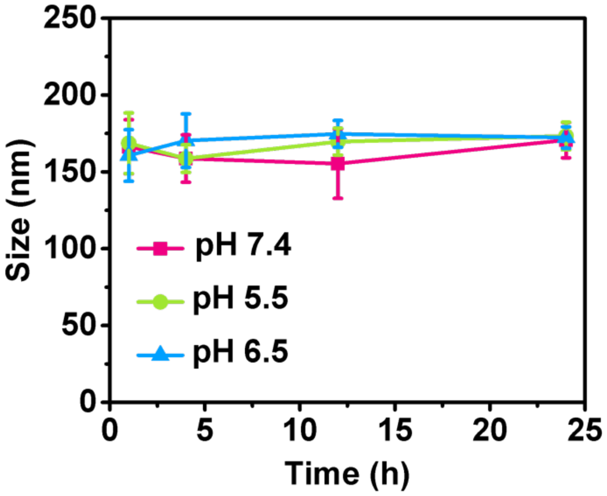

Supplementary Figure 12. Hydrodynamic diameter of Nano-BFF in PBS (pH = 7.4, 6.5, and 5.5) solutions for 1, 4, 12, and 24 h (n = 3 biologically independent samples). Data are presented as mean values ± SD.

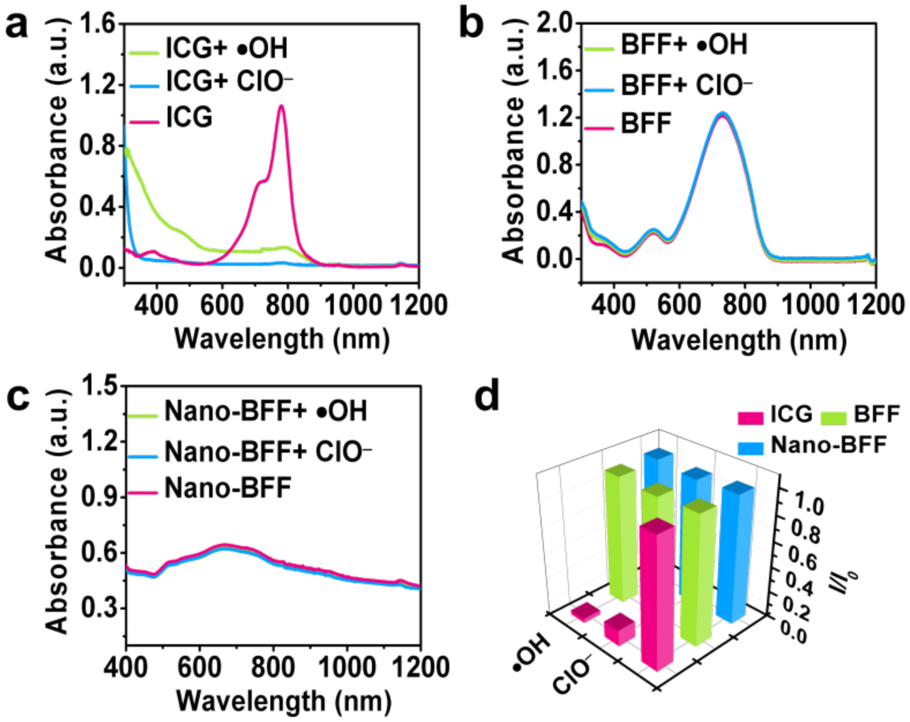

Supplementary Figure 13. Aggressive agent resistance of Nano-BFF. a-c UV-vis-NIR absorption spectra of **a** ICG, **b** BFF, and **c** Nano-BFF before and after the addition (200 μM) of •OH and ClO^- for 2 min. **d** Plots of I/I_0 versus •OH or ClO^-. I and I_0 are the maximal absorbance of ICG, BFF or Nano-BFF in the presence and absence of •OH or ClO^-.

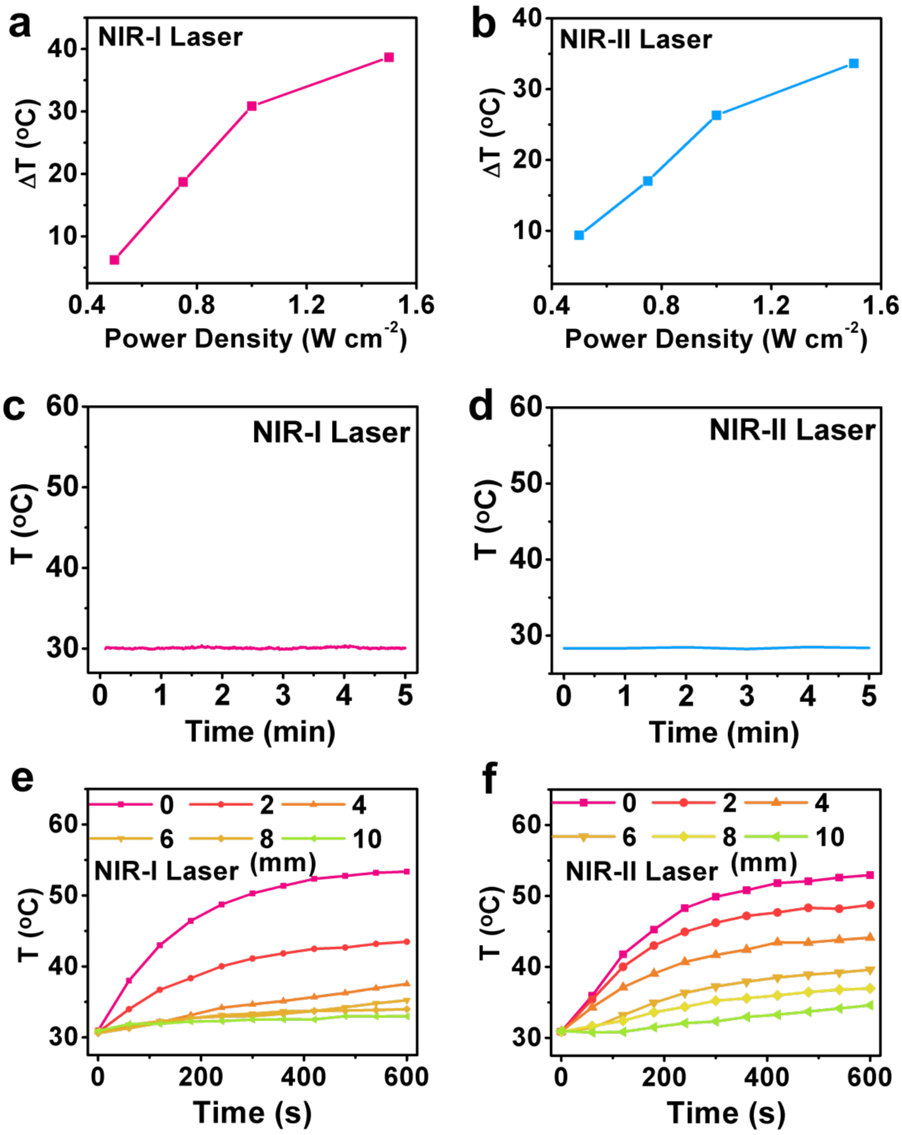

Supplementary Figure 14. Photothermal conversion performance of Nano-BFF. a,**b** Temperature increments of Nano-BFF after **a** 808 nm and **b** 1064 nm laser irradiation at different power densities. **c**,**d** Photothermal conversion curves of deionized water under **c** 808 nm and **d** 1064 nm laser irradiation. **e**,**f** Photothermal conversion curves of Nano-BFF aqueous solution upon exposure to **e** 808 nm and **f** 1064 nm laser irradiation under varied tissue thicknesses (0, 2, 4, 6, 8, and 10 mm).

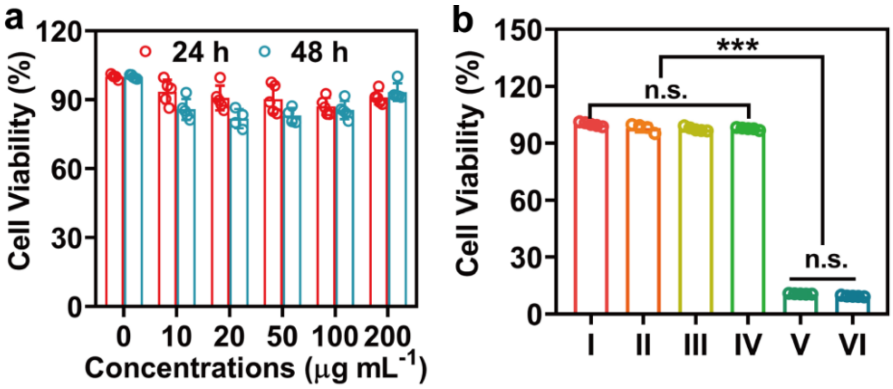

Supplementary Figure 15. Cellular cytotoxicity of Nano-BFF. a Cell viability of MCF-7 cells after incubation with different concentrations of Nano-BFF for 24 and 48 h (n = 5 biologically independent samples). **b** Cell viability of 4T1 cells after various treatments (I: control; II: NIR-I laser; III: NIR-II laser; IV: Nano-BFF; V: Nano-BFF + NIR-I laser; VI: Nano-BFF + NIR-II laser) (n = 5 biologically independent samples). Data are presented as mean values ± SD. n.s.: not significant. $p > 0.05$; *$p < 0.05$; **$p < 0.01$; ***$p < 0.001$, analyzed by Student's two-sided test.

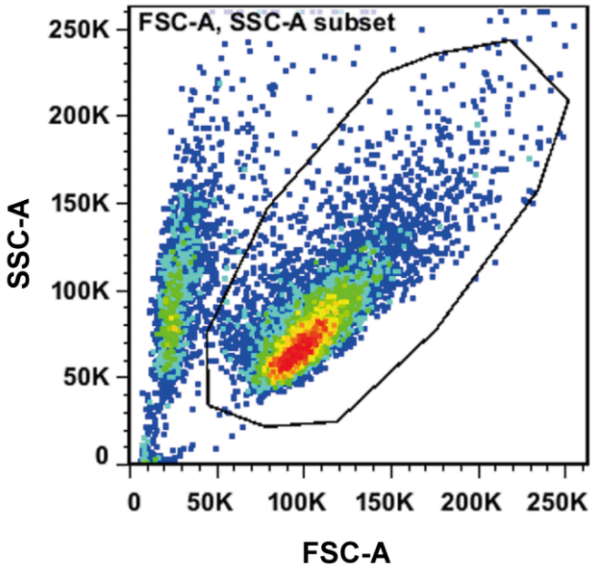

Supplementary Figure 16. Gating strategy for flow cytometric studies of 4T1 cells in Fig. 3h.

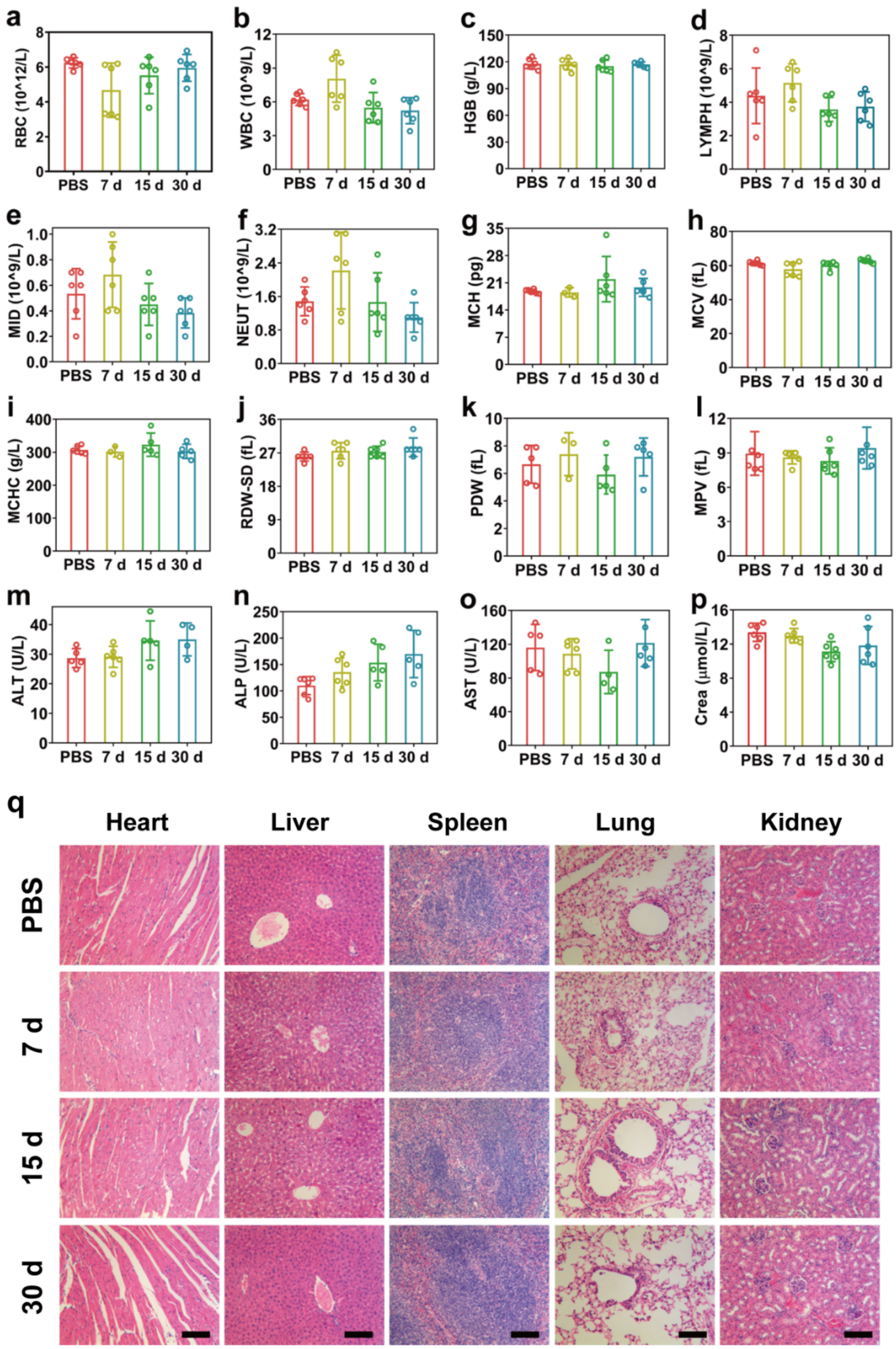

Supplementary Figure 17. In vivo toxicity evaluation of Nano-BFF. a-l Blood test parameters, including RBC, WBC, HGB, LYMPH, MID, NEUT, MCH, MCV, MCHC, RDW-SD, PDW, and MPV of the healthy Kunming mice after various treatments (n = 6 biologically

independent samples). **m-p** Blood biochemistry parameters, including ALT, ALP, AST, and Crea of the healthy Kunming mice after various treatments (n = 6 biologically independent samples). **q** H&E stained images acquired from the major organs (heart, liver, spleen, lung, and kidney) of Nano-BFF treated Kunming mice at the 7th, 15th, and 30th day post-injection. Scale bars: 100 μm. A representative image of three biological replicates from each group is shown.

Supplementary Note 1: Discussion for Supplementary Figure 17

Various blood indexes, including red blood cells (RBC), white blood cells (WBC), hemoglobin (HGB), lymphocyte (LYMPH), intermediate cell (MID), neutrophil (NEUT), mean corpuscular hemoglobin (MCH), mean corpuscular volume (MCV), mean corpuscular hemoglobin concentration (MCHC), red blood cell distribution width (RDW-SD), platelet distribution width (PDW), and mean platelet volume (MPV) were examined (Supplementary Fig. 17a-l). Additionally, the functional indicators associated with the liver and kidney of mice were tested, including alanine transaminase (ALT), alkaline phosphatase (ALP), aspartate transaminase (AST), and creatinine (Crea) (Supplementary Fig. 17m-p). All the markers of Nano-BFF treated groups exhibited no obvious difference with the PBS-treated group, demonstrating no negative impact on the blood biochemistry induced by Nano-BFF.

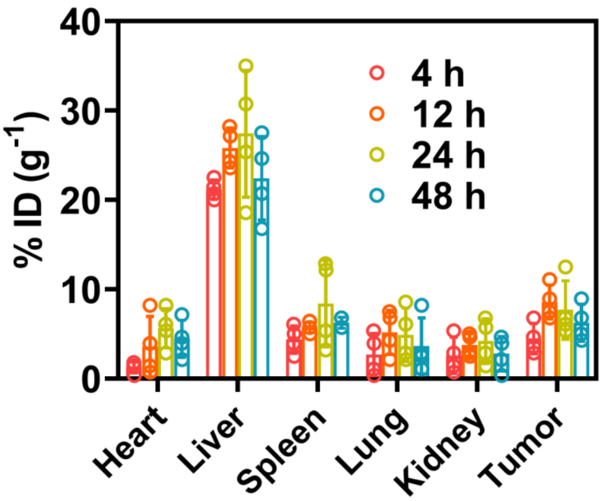

Supplementary Figure 18. In vivo biodistribution of Nano-BFF in major organs and tumors (ID% per tissue) after intravenous injection of Nano-BFF at various time points (4, 12, 24, and 48 h). n = 4 biologically independent samples. Data are presented as mean values ± SD.

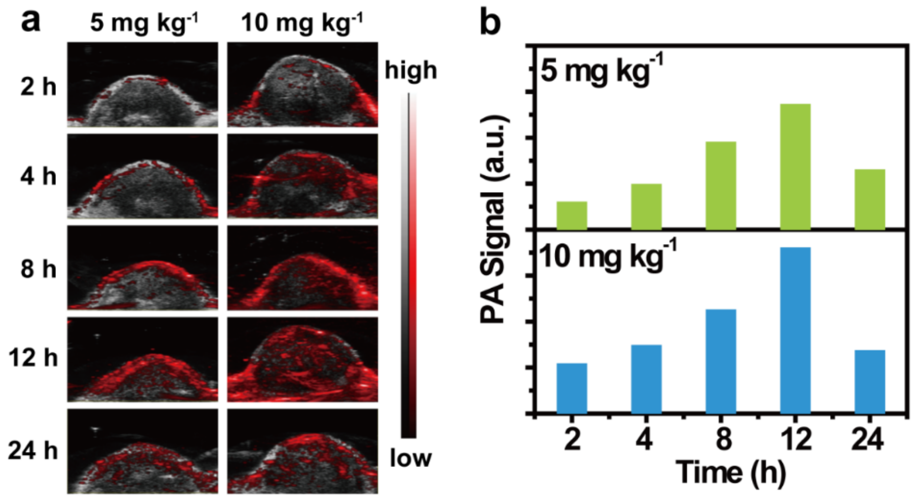

Supplementary Figure 19. **In vivo PA images of Nano-BFF. a** In vivo PA images of tumors under excitation at 900 nm under different concentrations (5 and 10 mg kg^{-1}), and **b** corresponding PA values after intravenous injection of Nano-BFF at various time points (2, 4, 8, 12, and 24 h).

Supplementary Note 2: Discussion for Supplementary Figure 19

By utilizing strong absorbance of Nano-BFF in both NIR-I and NIR-II biowindows, in vivo PA imaging analysis was performed to track the time-dependent distribution of Nano-BFF at post-injection. Different doses (5, 10, and 20 mg kg^{-1}) of Nano-BFF were intravenously injected into 4T1 tumor-bearing mice, and the PA images and intensities at tumor sites were recorded at various post-injection time points. As exhibited in Fig. 4f-h and Supplementary Fig. 19, the PA signal intensity increased gradually over time and reached its maximum at 12 h post-injection, confirming that the increased PA signal was resulted from the efficient accumulation of Nano-BFF.

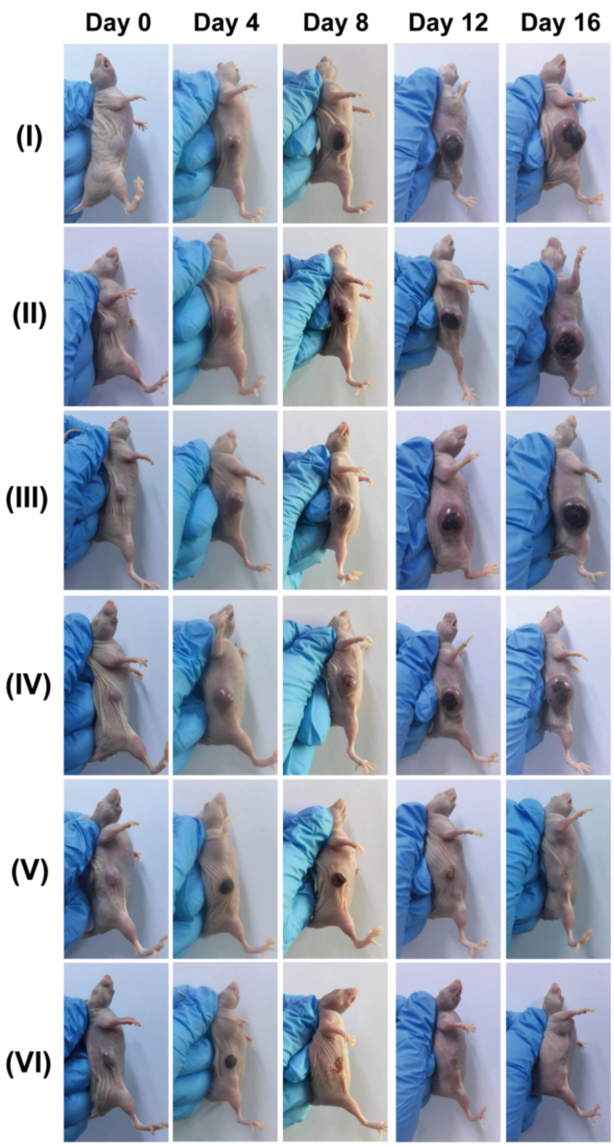

Supplementary Figure 20. In vivo photonic hyperthermia of Nano-BFF. Representative photographs of 4T1 tumor bearing mice after various treatments (I: control; II: NIR-I laser; III: NIR-II laser; IV: Nano-BFF; V: Nano-BFF + NIR-I laser; VI: Nano-BFF + NIR-II laser).

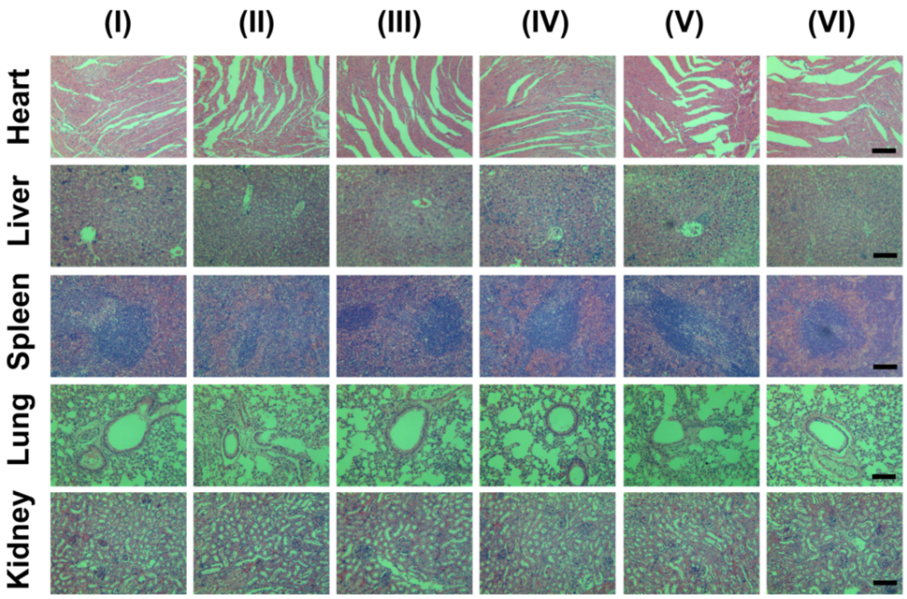

Supplementary Figure 21. Biosafety of Nano-BFF after various treatments. H&E staining of major organs from different treatment groups (I: control; II: NIR-I laser; III: NIR-II laser; IV: Nano-BFF; V: Nano-BFF + NIR-I laser; VI: Nano-BFF + NIR-II laser). Scale bars: 100 μm. A representative image of three biological replicates from each group is shown.

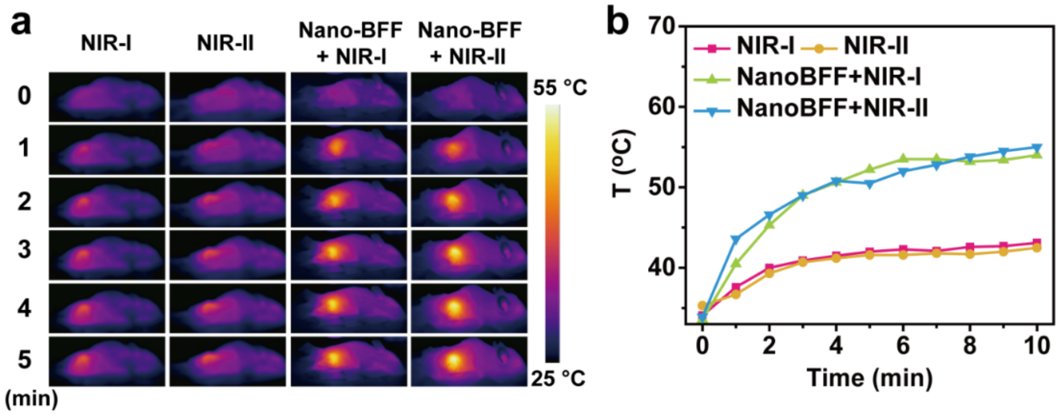

Supplementary Figure 22. **IR thermal images and temperature variations**. **a** IR thermal images, and **b** temperature variations at the tumor sites of 4T1 tumor-bearing BALB/c mice in various treatment groups, including NIR-I laser, NIR-II laser, Nano-BFF + NIR-I laser, and Nano-BFF + NIR-II laser groups (n = 5 biologically independent samples).

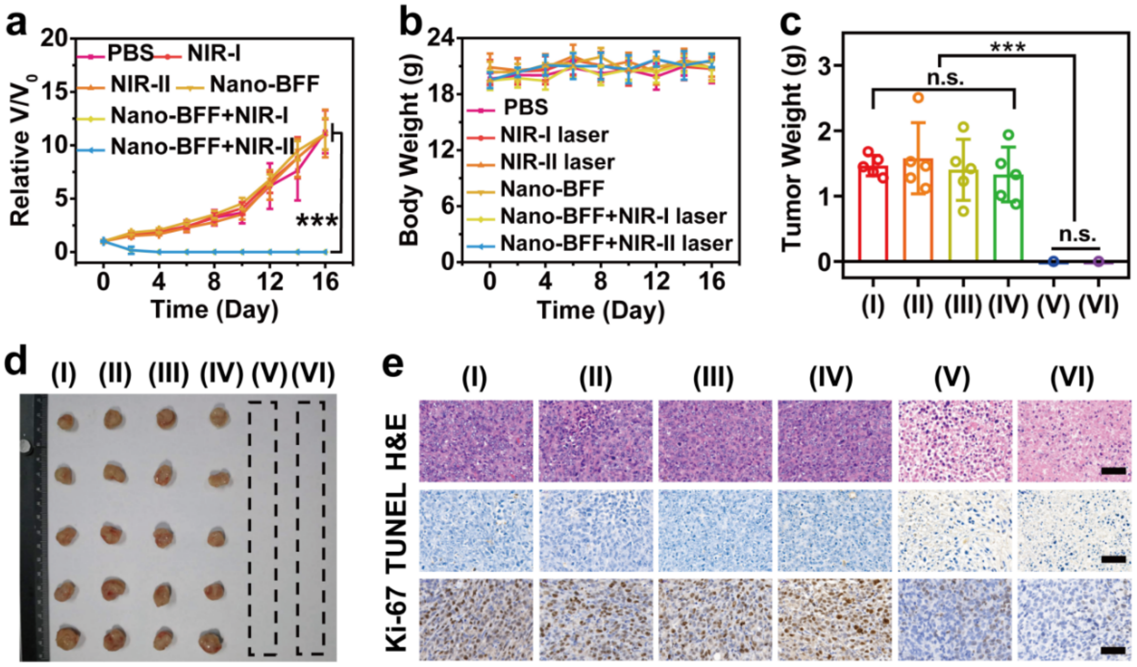

Supplementary Figure 23. Relative tumor volumes, body weights of the mice, tumor weights, photographs of tumors, and staining of tumor tissues. a Relative tumor volumes, **b** body weights of the mice, **c** tumor weights, and **d** photographs of tumors in different treatment groups (n = 5 biologically independent samples). **e** H&E, TUNEL, and Ki-67 staining of tumor tissues in various treatment groups (I: PBS; II: NIR-I laser; III: NIR-II laser; IV: Nano-BFF; V: Nano-BFF + NIR-I laser; VI: Nano-BFF + NIR-II laser). Scale bars: 50 μm. Data are presented as mean values ± SD. n.s.: not significant. $p > 0.05$; $^{*}p < 0.05$; $^{**}p < 0.01$; $^{***}p < 0.001$, analyzed by Student's two-sided test. A representative image of three biological replicates from each group is shown in **e**.

Supplementary Note 3: Discussion for Supplementary Figure 22 and 23

Immune competent 4T1 tumor-bearing BALB/c mouse model was established to evaluate the photonic hyperthermia effect of Nano-BFF in vivo. The mice were randomly divided into 6 groups: PBS (control), NIR-I laser, NIR-II laser, Nano-BFF, Nano-BFF + NIR-I laser, and Nano-BFF + NIR-II laser. The tumors were exposed to laser irradiation (808 nm or 1064 nm) at 12 h post-injection for 10 min. Real-time temperature and in-situ thermal images were monitored by an infrared imaging system to visualize the temperature elevation under NIR-I or NIR-II laser irradiation (Supplementary Fig. 22a). As presented in Supplementary Fig. 22b, the

temperature of Nano-BFF + NIR-I laser and Nano-BFF + NIR-II laser groups elevated quickly by 19.9 °C and 18.1 °C during the first 6 min and then increased slowly to 55.1 °C and 54.0 °C within 10 min, respectively. In contrast, the groups of NIR-I laser and NIR-II laser exhibited slight increase in temperature, with the final temperature of 43.1 °C and 42.5 °C respectively, which demonstrated that Nano-BFF exhibited excellent capability in elevating tumor temperature upon NIR-I laser or NIR-II laser irradiation. Tumor volumes and body weights in all treatment groups were recorded every 2 day. The groups with the injection of PBS and Nano-BFF without laser exposure revealed rapid tumor growth during the treatment period. In comparison, mice administrated with Nano-BFF and subsequent NIR-I laser or NIR-II laser irradiation achieved complete tumor suppression, validating that Nano-BFF had high therapeutic efficacy in vivo in both NIR-I and NIR-II biowindows (Supplementary Fig. 23 a,c,d). In addition, no significant body weight loss of mice could be observed in all treatment groups, implying high biosafety of Nano-BFF in vivo (Supplementary Fig. 23b).

To further validate the enhanced therapeutic efficacy of Nano-BFF, the representative tumors of the mice in all treatment groups were collected for histological analysis using H&E, TUNEL, and Ki-67 antibody staining. Large areas of necrosis/apoptosis were observed on the tumor slices for the groups of Nano-BFF + NIR-I laser and Nano-BFF + NIR-II laser, which were not obvious for control groups of PBS, NIR-I laser, NIR-II laser, and Nano-BFF (Supplementary Fig. 23e). These results further verified prominent in vivo photothermal therapeutic efficacy of Nano-BFF.

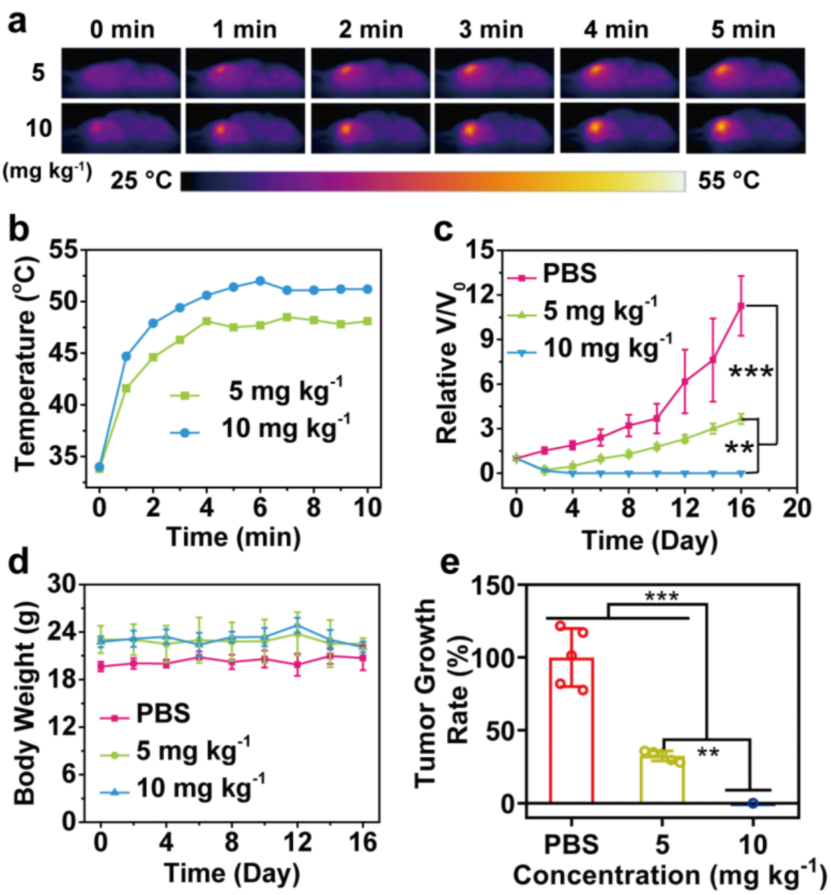

Supplementary Figure 24. IR thermal images, temperature variations, relative tumor volumes, body weights of the mice, and tumor growth rates. **a** IR thermal images, and **b** temperature variations at the tumor sites of the mice after different treatments. **c** Relative tumor volumes, **d** body weights of the mice, and **e** tumor growth rates in different treatment groups, including Nano-BFF (5 mg kg^{-1}) + NIR-II laser, and Nano-BFF (10 mg kg^{-1}) + NIR-II laser groups (n = 5 biologically independent samples). Data are presented as mean values ± SD. $p > 0.05$; *$p < 0.05$; **$p < 0.01$; ***$p < 0.001$, analyzed by Student's two-sided test.

Supplementary Note 4: Discussion for Supplementary Figure 24

To evaluate the in vivo antitumor efficacy in the NIR-II biowindow, various doses of Nano-BFF were intravenously injected into 4T1 tumor bearing mice at 12 h post-injection. As showed in Supplementary Fig. 24a,b, the tumor temperature increased by 14.1 and 17.2 °C at the injection doses of 5 and 10 mg kg^{-1} under NIR-II laser irradiation respectively, indicating excellent hyperthermia effect from Nano-BFF upon NIR-II laser exposure. Importantly, Nano-BFF (5 mg kg^{-1}) plus NIR-II laser exposure caused the tumor suppression with the tumor growth

rate of 67.6%. Complete tumor ablation without further recurrence was observed for the mice received with Nano-BFF (10 mg kg^{-1}) administration and NIR-II laser irradiation (Supplementary Fig. 24c,e). In addition, no significant body weight variation was detected in all treatment groups, demonstrating negligible side effect of Nano-BFF toward the mouse health (Supplementary Fig. 24d).

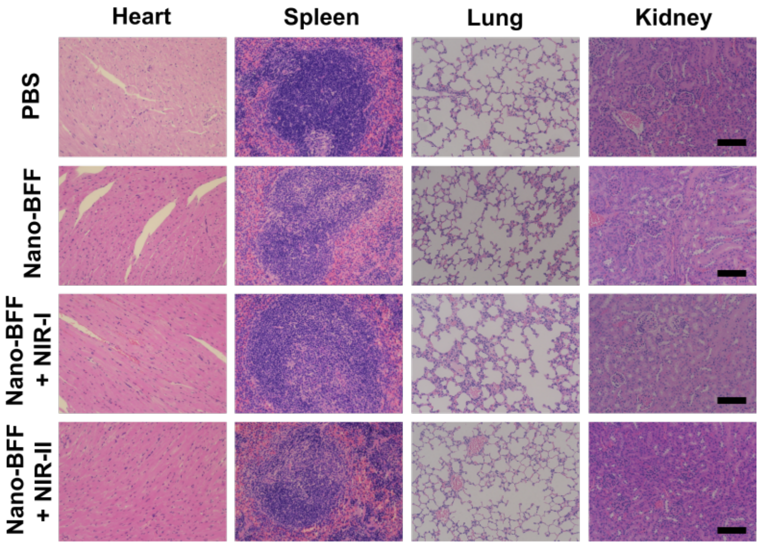

Supplementary Figure 25. H&E staining images of major organs from orthotopic liver tumor bearing mice in various treatment groups, including PBS, Nano-BFF, Nano-BFF + NIR-I laser, and Nano-BFF + NIR-II laser groups. Scale bars: 100 μm. A representative image of three biological replicates from each group was shown.
