# Supplementary material for: Self-assembled organic nanomedicine enables ultrastable photo-to-heat converting theranostics in the second near-infrared biowindow
Source: Nat Commun. 2021 Jan 11;12:218. doi: 10.1038/s41467-020-20566-6 (PMC7801739; doi:10.1038/s41467-020-20566-6)
Supplement: Supplementary file 3 — Reporting Summary [file 41467_2020_20566_MOESM3_ESM.pdf]

nature research

Corresponding author(s): Yu Chen and Yanli Zhao

Last updated by author(s): Nov 25, 2020

Reporting Summary

Nature Research wishes to improve the reproducibility of the work that we publish. This form provides structure for consistency and transparency in reporting. For further information on Nature Research policies, see our Editorial Policies and the Editorial Policy Checklist.

Statistics

For all statistical analyses, confirm that the following items are present in the figure legend, table legend, main text, or Methods section.

n/a	Confirmed	
[ ]	[x]	The exact sample size (n) for each experimental group/condition, given as a discrete number and unit of measurement
[ ]	[x]	A statement on whether measurements were taken from distinct samples or whether the same sample was measured repeatedly
[ ]	[x]	The statistical test(s) used AND whether they are one- or two-sided<br>*Only common tests should be described solely by name; describe more complex techniques in the Methods section.*
[x]	[ ]	A description of all covariates tested
[x]	[ ]	A description of any assumptions or corrections, such as tests of normality and adjustment for multiple comparisons
[ ]	[x]	A full description of the statistical parameters including central tendency (e.g. means) or other basic estimates (e.g. regression coefficient) AND variation (e.g. standard deviation) or associated estimates of uncertainty (e.g. confidence intervals)
[ ]	[x]	For null hypothesis testing, the test statistic (e.g. F, t, r) with confidence intervals, effect sizes, degrees of freedom and P value noted<br>*Give P values as exact values whenever suitable.*
[x]	[ ]	For Bayesian analysis, information on the choice of priors and Markov chain Monte Carlo settings
[x]	[ ]	For hierarchical and complex designs, identification of the appropriate level for tests and full reporting of outcomes
[x]	[ ]	Estimates of effect sizes (e.g. Cohen's d, Pearson's r), indicating how they were calculated

Our web collection on statistics for biologists contains articles on many of the points above.

Software and code

Policy information about availability of computer code

Data collection	TEM (JEM-2100F, JEOL), UV-vis-NIR (UV-3600, Shimadzu), Infrared Thermal Camera (FLIR A325SC), Confocal Microscopy (FV1000, Olympus), FV10-ASW 1.7 Viewer,Flow cytometry (FACSCalibur, BD Bioscience), PA imaging system (Vevo LAZR, VisualSonics), ChemBioDraw Ultra 12.0.
Data analysis	Flow cytometry analysis was performed using FlowJo software package (FlowJo 7.6.1), GraphPad Prism 8, OriginPro 2016, ImageJ 1.43u, Microsoft Excel 2016.

For manuscripts utilizing custom algorithms or software that are central to the research but not yet described in published literature, software must be made available to editors and reviewers. We strongly encourage code deposition in a community repository (e.g. GitHub). See the Nature Research guidelines for submitting code & software for further information.

Data

Policy information about availability of data

All manuscripts must include a data availability statement. This statement should provide the following information, where applicable:

- Accession codes, unique identifiers, or web links for publicly available datasets
- A list of figures that have associated raw data
- A description of any restrictions on data availability

The authors declare that the data supporting the findings of this study are available within the paper and its supplementary information files. The data are also available from the authors upon reasonable request.

Field-specific reporting

Please select the one below that is the best fit for your research. If you are not sure, read the appropriate sections before making your selection.

☒ Life sciences ☐ Behavioural & social sciences ☐ Ecological, evolutionary & environmental sciences

For a reference copy of the document with all sections, see nature.com/documents/nr-reporting-summary-flat.pdf

Life sciences study design

All studies must disclose on these points even when the disclosure is negative.

Sample size	Sample size was determined based on the minimum number of animals/replicates and was described in figure legend.
Data exclusions	No data was excluded from this study.
Replication	All experiments were performed with independent replicates as described in the figure legends.
Randomization	Described in "Methods". The cells were randomly seeded in a 96-well/12-well or 6 well plates and animals were randomly divided into different groups.
Blinding	The investigators were blinded to group allocation during data collection and/or analysis.

Reporting for specific materials, systems and methods

We require information from authors about some types of materials, experimental systems and methods used in many studies. Here, indicate whether each material, system or method listed is relevant to your study. If you are not sure if a list item applies to your research, read the appropriate section before selecting a response.

Materials & experimental systems

n/a	Involved in the study	
☐	☒	Antibodies
☐	☒	Eukaryotic cell lines
☒	☐	Palaeontology and archaeology
☐	☒	Animals and other organisms
☒	☐	Human research participants
☒	☐	Clinical data
☒	☐	Dual use research of concern

Methods

n/a	Involved in the study	
☒	☐	ChIP-seq
☐	☒	Flow cytometry
☒	☐	MRI-based neuroimaging

Antibodies

Antibodies used	Anti-Ki-67 Antibody, catalog n.ab15580, abcam
Validation	Anti-Ki-67 Antibody was used according to the manufacturers' instruction.

Eukaryotic cell lines

Policy information about cell lines

Cell line source(s)	4T1 and MCF-7 breast cancer cells were obtained from Shanghai Institutes for Biological Sciences (SIBS), Shanghai, China
Authentication	The cell line we used was morphologically confirmed according to the information provided by SIBS.
Mycoplasma contamination	All cell lines were tested for mycoplasma contamination. No mycoplasma contamination was found.
Commonly misidentified lines (See ICLAC register)	No commonly misidentified cells lines were used.

Animals and other organisms

Policy information about studies involving animals; ARRIVE guidelines recommended for reporting animal research

Laboratory animals	Balb/c nude mice (female, 5-6 weeks), Balb/c mice (female, 5-6 weeks), and Kunming mice (female, 4-5 weeks) were purchased from Shanghai SLAC Laboratory animal Co., Ltd.

Wild animals	This study did not involve wild animals.
Field-collected samples	This study did not involve samples collected from field.
Ethics oversight	All animal experiment methods were in accordance with the regulations of the Regional Ethics Committee for Animal Experiments approved by the Administrative Committee of Laboratory Animals of of Shanghai Tenth People's Hospital, Tongji University School of Medicine.

Note that full information on the approval of the study protocol must also be provided in the manuscript.

Flow Cytometry

Plots

Confirm that:

- [x] The axis labels state the marker and fluorochrome used (e.g. CD4-FITC).
- [x] The axis scales are clearly visible. Include numbers along axes only for bottom left plot of group (a 'group' is an analysis of identical markers).
- [x] All plots are contour plots with outliers or pseudocolor plots.
- [x] A numerical value for number of cells or percentage (with statistics) is provided.

Methodology

Sample preparation	After various treatments, 4T1 cells were gently washed with PBS and collected by trypsinization and centrifugation. Then, the cells were resuspended in PBS and labeled with Annexin V-FITC and PI for flow cytometry test.
Instrument	BD FACSCalibur, BD Bioscience
Software	FlowJo software package (version 7.6.1)
Cell population abundance	The absolute cells around 10000 cells were analyzed for fluorescent intensity in the defined gate.
Gating strategy	Living cells were selected by FSC and SSC analysis. Green and red fluorescence of Annexin V-FITC and PI in cells were detected by FITC and PI channel.

- [x] Tick this box to confirm that a figure exemplifying the gating strategy is provided in the Supplementary Information.
